# Isolated Congenital Absence of Lumbosacral Facet Joint

**DOI:** 10.1155/2019/1465460

**Published:** 2019-02-12

**Authors:** Joseph Maalouly, Dany Aouad, Hicham G. Abd El Nour, Alexandre Nehme, Fouad Jabbour

**Affiliations:** Department of Orthopedic Surgery and Traumatology, Saint George University Medical Center, Balamand University, P.O. Box 166378, Achrafieh, Beirut 1100 2807, Lebanon

## Abstract

A congenital absence of the lumbar facet joint is a rare reported condition. This is the case of a 32-year-old male patient presenting with severe low back pain radiating to his right lower limb. Physical examination revealed no motor deficits, but right lower limb numbness was reported over dorsum aspect of the right foot. No other sensory or motor disturbances were reported or found on exam. Imaging studies, consisting of a spine MRI and spine CT scan with 3D reconstruction, revealed the absence of the right L5-S1 zygapophyseal joint. The patient was treated conservatively with resolution of his symptoms on one-month follow-up.

## 1. Introduction

A congenital absence of the unilateral lumbar L5 inferior articular process is a rare spinal anomaly, with about 25 reported cases found in the literature [1, 2]. In around 80% of the reported cases, the L5-S1 joint was affected [3]. The etiology is presumed to be due to a dysfunction of the vertebral ossification centers during fetal life, which consist of two centers in the neural arches and one center in the vertebral body [4, 5]. Surgical intervention consisting of spinal fusion is rarely performed [4], and most cases are resolved with conservative treatment, especially in patients with no instability. We report a case of unilateral absence of the fifth lumbar inferior articular process with a review of the relevant literature.

## 2. Case Report

A 32-year-old male patient presented to our emergency department complaining of severe low back pain radiating to the right lower limb. He was an obese man with a previous acute back pain episode few months ago. He had previously experienced back pain after hard work or exercise for several years. The findings of a physical examination were unremarkable, except for mild tenderness at the right lumbar paravertebral area and right lower limb numbness over dorsum aspect of the right foot. Motor power at the level of the extensor hallucis longus was 5/5, triceps surae was 5/5, right flexor hallucis longus was 5/5, and tibialis anterior was 5/5. No pathological reflexes were noted. The straight leg raising test was positive on the right.

**Figure 1:**
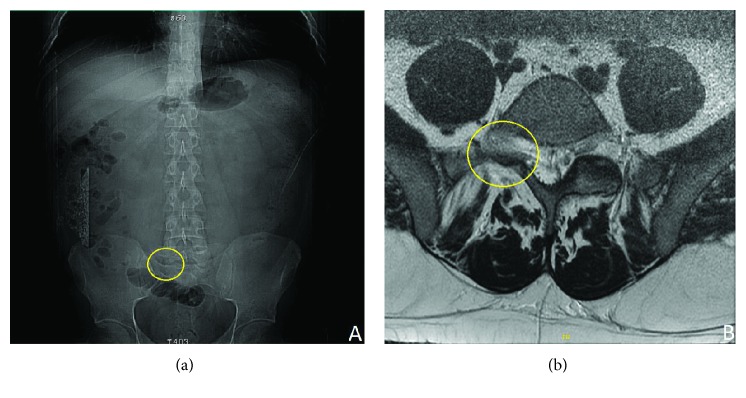
(a) Scout X-ray image showing the absence of the right L5-S1 zygapophyseal joint. (b) On axial spine MRI, the right articular process of S1 is absent, with the inferior articular process of the L5 markedly hypoplastic.

Initially, plain radiographs followed by a spine MRI were ordered which showed the absence of the right L5-S1 zygapophyseal joint (Figure 1).

MRI confirmed the absence of the unilateral L5 lumbar inferior articular process. However, the contralateral joint was normal and did not show any pathological changes. There was a disc herniation seen at the L5-S1 level.

**Figure 2:**
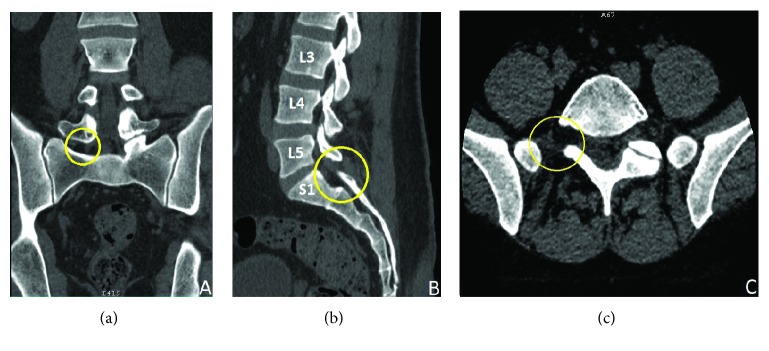
(a) Coronal cut of spine CT scan showing the absence of the right L5-S1 zygapophyseal joint forming a defect. (b) Sagittal cut of spine CT scan showing the absence of the L5 inferior articular process on the right with normal neighboring joints. (c) Axial CT scan cut of the spine further confirming the absence of the L5-S1 facet joint on the right side.

**Figure 3:**
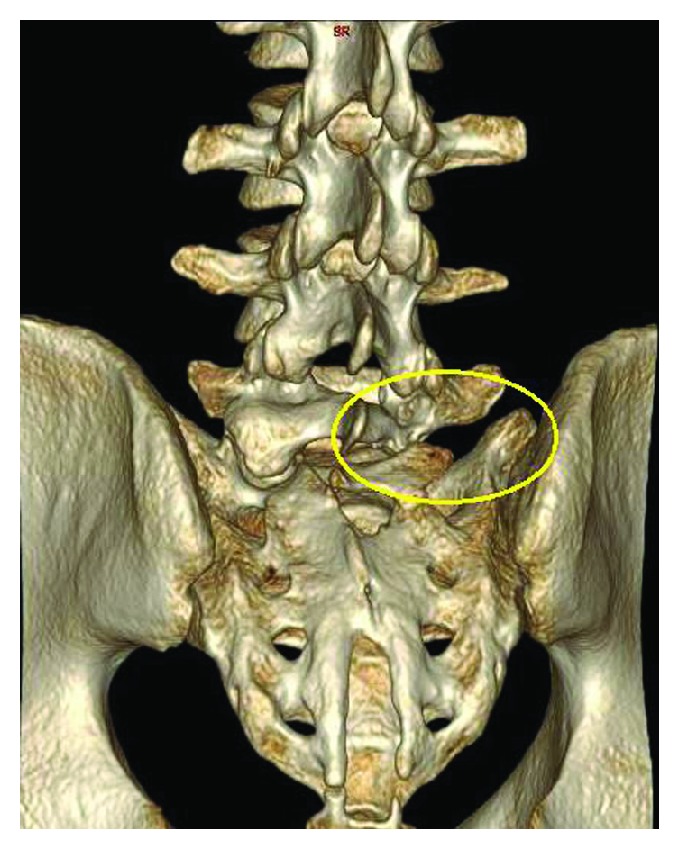
CT scan 3D reconstruction shows agenesis of the L5 inferior articular process on the right side with no other related bony abnormalities identified.

On spine CT scan (Figure 2) along with 3D reconstruction (Figure 3), a better view of the affected area was seen, showing a detailed visual of the bony anomaly.

After thorough clinical examination and relevant imaging, the patient was managed conservatively with IV medications and discharged home on supportive treatment with anti-inflammatory modalities. He was scheduled for a follow-up appointment for his symptoms.

## 3. Discussion

Congenital absence of the L5 zygapophyseal joint is a rare abnormality.

The exact etiology is still unknown but the accepted explanation is as follows: there are three primary ossification centers in each vertebra: two centers in the neural arches and one center in the vertebral body. The facet defect is thought to be caused by failure of these ossification processes during fetal life [4–6]. It is postulated that this failure is due to insufficient blood supply during fetal life [4].

Although it is rare for this anomaly to cause any symptoms, cases of lower limb weakness caused by spinal instability are previously reported. However, in most of these reported cases, almost 90% are successfully treated conservatively. Only in severe cases was surgical spinal fusion needed to achieve stability. Some of these reported cases have been associated with conjoined nerve roots; however, they are rarely reported [1]. Rask et al. linked congenital osseous defects of the lumbosacral spine to the presence of root anomalies [7], yet it is possible to have isolated osseous defects, such as in this case. Because of the small number of cases, treatment remains controversial, and each case should be treated individually depending on the severity of the symptoms, the neurological involvement, and on the presence of surgical indications. Despite that, no specific guidelines are currently published for the treatment algorithm of such conditions.

In summary, we report a rare case of congenital absence of the right L5 inferior articular process who presented for low back pain radiating to the right lower limb, successfully managed conservatively with regular follow-up. Surgeons should be aware of the possible absence of the lumbosacral zygapophyseal joint when a patient shows an unusual clinical presentation, and/or congenital osseous anomaly, especially in younger persons [8].
